# Evaluation of an integrated knowledge translation approach used for updating the Cochrane Review of Patient Decision Aids: a pre-post mixed methods study

**DOI:** 10.1186/s40900-024-00550-w

**Published:** 2024-02-09

**Authors:** Krystina B. Lewis, Maureen Smith, Dawn Stacey, Meg Carley, Ian D. Graham, Cochrane Review of Patient Decision Aids Research Team, Robert J. Volk, Elisa E. Douglas, Lissa Pacheco-Brousseau, Jeanette Finderup, Janet Gunderson, Michael J. Barry, Carol L. Bennett, Paulina Bravo, Karina Dahl Steffensen, Amédé Gogovor, Shannon E. Kelly, France Légaré, Henning Søndergaard, Logan Trenaman, Lyndal Trevena

**Affiliations:** 1 School of Nursing, Faculty of Health Sciences, University of Ottawa, Ottawa, ON Canada; 2 Knowledge User, Ottawa, ON Canada; 3 Cochrane Consumer, Ottawa, ON Canada; 4 Ottawa Hospital Research Institute, Ottawa, ON Canada; 5 School of Epidemiology and Public Health, University of Ottawa, Ottawa, ON Canada

**Keywords:** Co-production, Integrated knowledge translation, Knowledge users, Knowledge mobilization, Knowledge co-creation, Patient/consumers, Systematic review

## Abstract

**Background:**

When people who can use or benefit from research findings are engaged as partners on study teams, the quality and impact of findings are better. These people can include patients/consumers and clinicians who do not identify as researchers. They are referred to as “knowledge users”. This partnered approach is called integrated knowledge translation (IKT). We know little about knowledge users’ involvement in the conduct of systematic reviews. We aimed to evaluate team members’ degree of meaningful engagement and their perceptions of having used an IKT approach when updating the Cochrane Review of Patient Decision Aids.

**Methods:**

We conducted a pre-post mixed methods study. We surveyed all team members at two time points. Before systematic review conduct, all participating team members indicated their preferred level of involvement within each of the 12 steps of the systematic review process from “Screen titles/abstracts” to “Provide feedback on draft article”. After, they reported on their degree of satisfaction with their achieved level of engagement across each step and the degree of meaningful engagement using the Patient Engagement In Research Scale (PEIRS-22) across 7 domains scored from 100 (extremely meaningful engagement) to 0 (no meaningful engagement). We solicited their experiences with the IKT approach using open-ended questions. We analyzed quantitative data descriptively and qualitative data using content analysis. We triangulated data at the level of study design and interpretation.

**Results:**

Of 21 team members, 20 completed the baseline survey (95.2% response rate) and 17/20 (85.0% response rate) the follow-up survey. There were 11 (55%) researchers, 3 (15%) patients/consumers, 5 (25%) clinician-researchers, and 1 (5%) graduate student. At baseline, preferred level of involvement in the 12 systematic review steps varied from n = 3 (15%) (search grey literature sources) to n = 20 (100%) (provide feedback on the systematic review article). At follow-up, 16 (94.1%) participants were totally or very satisfied with the extent to which they were involved in these steps. All (17, 100%) agreed that the process was co-production. Total PEIRS-22 scores revealed most participants reported extremely (13, 76.4%) or very (2, 11.8%) meaningful degree of engagement. Triangulated data revealed that participants indicated benefit to having been engaged in an authentic research process that incorporated diverse perspectives, resulting in better and more relevant outputs. Reported challenges were about time, resources, and the logistics of collaborating with a large group.

**Conclusion:**

Following the use of an IKT approach during the conduct of a systematic review, team members reported high levels of meaningful engagement. These results contribute to our understanding of ways to co-produce systematic reviews.

**Supplementary Information:**

The online version contains supplementary material available at 10.1186/s40900-024-00550-w.

## Abstract (plain language summary)

When people who can use or benefit from research findings are engaged as partners on study teams, the quality and impact of findings are better. These people can include patients/consumers and clinicians who do not identify as researchers. This partnered approach is called *integrated knowledge translation* (IKT). This approach is rarely used and there is little information about using it with systematic reviews. A systematic review is a type of study that provides the best available evidence on a given topic by combining data from all existing studies. The aim of this study was to find out how engaged our team members felt when partnering on our systematic review about patient decision aids. Twenty of 21 team members participated in the study, including 11 researchers, 3 patients/consumers, 5 clinician-researchers, and 1 graduate student. We asked our team members to complete a survey about their experience as part of our IKT research process at two time points: before starting the study and after the study was done. Most team members felt extremely or very engaged in the process. All team members felt like partners. They gave examples of how this was achieved. Advantages to using the IKT approach included knowledge sharing, inclusion of more diverse voices, a more authentic research process, better and more relevant results, and personal benefits (e.g. enjoyment from being involved). Disadvantages to using this approach was that it took more time and resources. Three team members said there were no disadvantages. It is possible for patients/consumers and clinicians to partner and feel engaged with research teams doing systematic reviews. Our findings may help researchers engage knowledge users as equal partners on study teams.

**Supplementary Information:**

The online version contains supplementary material available at 10.1186/s40900-024-00550-w.

## Introduction

Effective co-produced research can increase the quality, relevance, uptake and impact of research [1–6]. Research co-production requires researchers to partner with diverse individuals who are positioned and interested in using or benefiting from the research findings as equal members of the research team [7, 8]. These individuals are referred to as knowledge users and may include patients, caregivers/family members, clinicians/health care professionals, the public, and policymakers [8]. At the core of successful co-produced research are genuine partnerships with meaningfully engaged team members. Hamilton et al. define meaningful engagement as “*the planned, supported, and valued involvement of team members in the research process within a positive environment in which they contribute and have a rewarding experience*” [9, 10]. To achieve meaningful engagement means countering the risks associated with co-production including tokenism, power imbalances, questioning reasons for engagement, transactional relationships where only one party benefits, and suboptimal preparation and training for knowledge users’ roles on the team [1, 10–12].

Integrated knowledge translation (IKT) is an approach that falls under the research co-production umbrella. It aims for meaningful engagement of researcher and knowledge user team members throughout the research process [13]. In IKT “*researchers work with knowledge users who identify a problem and have the authority to implement the research recommendations*” [14] (p. 299) with the goal of co-producing findings that have real-world application. Teams who use IKT are committed to collaboration, engagement, and the exchange of knowledge (e.g., current evidence, lived experience, clinical experience, contextual, sociopolitical influences) with opportunities for all parties to participate at any step: from the conception of the study design to its conduct, data collection, data synthesis and interpretation, and the dissemination and application of findings [15]. In many countries, funding agencies are increasingly supporting—some even mandating—knowledge users’ involvement regardless of topic or study design [16]. Systematic reviews are no exception, particularly as the synthesized findings are intended to provide decision-makers with the highest quality and most current evidence needed to make practice and policy decisions to improve care and patient outcomes [17]. Patients, caregivers, and the public also use systematic reviews to make decisions about their own health care. In fact, an increasing number of organizations dedicated to the conduct of knowledge syntheses either mandate or strongly recommend knowledge user engagement in review conduct and offer guidance. For example, the Cochrane Collaboration’s commitment to this is detailed in their 2022-published *Cochrane consumer engagement and involvement framework to 2027* [18]; the Joanna Briggs Institute scoping review methodology group also recently published guidance for co-creation with knowledge users [19].

There are increased calls to evaluate co-production approaches in health research, in particular systematic reviews. Thus, we aimed to evaluate team members’ degree of meaningful engagement throughout the conduct of a systematic review. We also aimed to explore their perspectives on the extent to which the research process was one of co-production, and the benefits and challenges of using an IKT approach for conducting a systematic review.

## Methods

### Study design

We conducted a pre-post mixed methods study to evaluate our IKT approach used within the conduct of a systematic review. The Ottawa Health Science Network Research Ethics Board (#20220107-01H) and the University of Ottawa Research Ethics Board (#H-03-22-7991) approved the study. We report our findings using the Strengthening the Reporting of Observational Studies in Epidemiology (STROBE) guidelines [20].

### Context and setting

The most recent update of the Cochrane Review on Patient Decision Aids﻿ served as the context for this IKT evaluation study. Patient decision aids (PtDAs) are evidence-based knowledge translation tools that are designed to engage patients in making informed and values-based health decisions [21, 22]. The IPDAS Collaboration strongly recommends the deliberate involvement of knowledge users in PtDA development, including patients, family members, clinicians, even professional organizations, to ensure developed PtDAs meet their needs [23]. Involvement of knowledge users in PtDA development is associated with successful implementation of PtDAs in clinical practice [24]. The motivations underlying knowledge-user involvement in PtDA co-development can be likened to the aims of IKT and research co-production: by engaging users of the products/findings from the beginning, they are more likely to be useful and relevant to the real-world. With the proliferation of trials of PtDAs for people facing health care decisions, our international team of researchers and knowledge users received funding from the CIHR to update the Cochrane Systematic Review of PtDAs using an IKT approach [25].

**Fig. 1:**
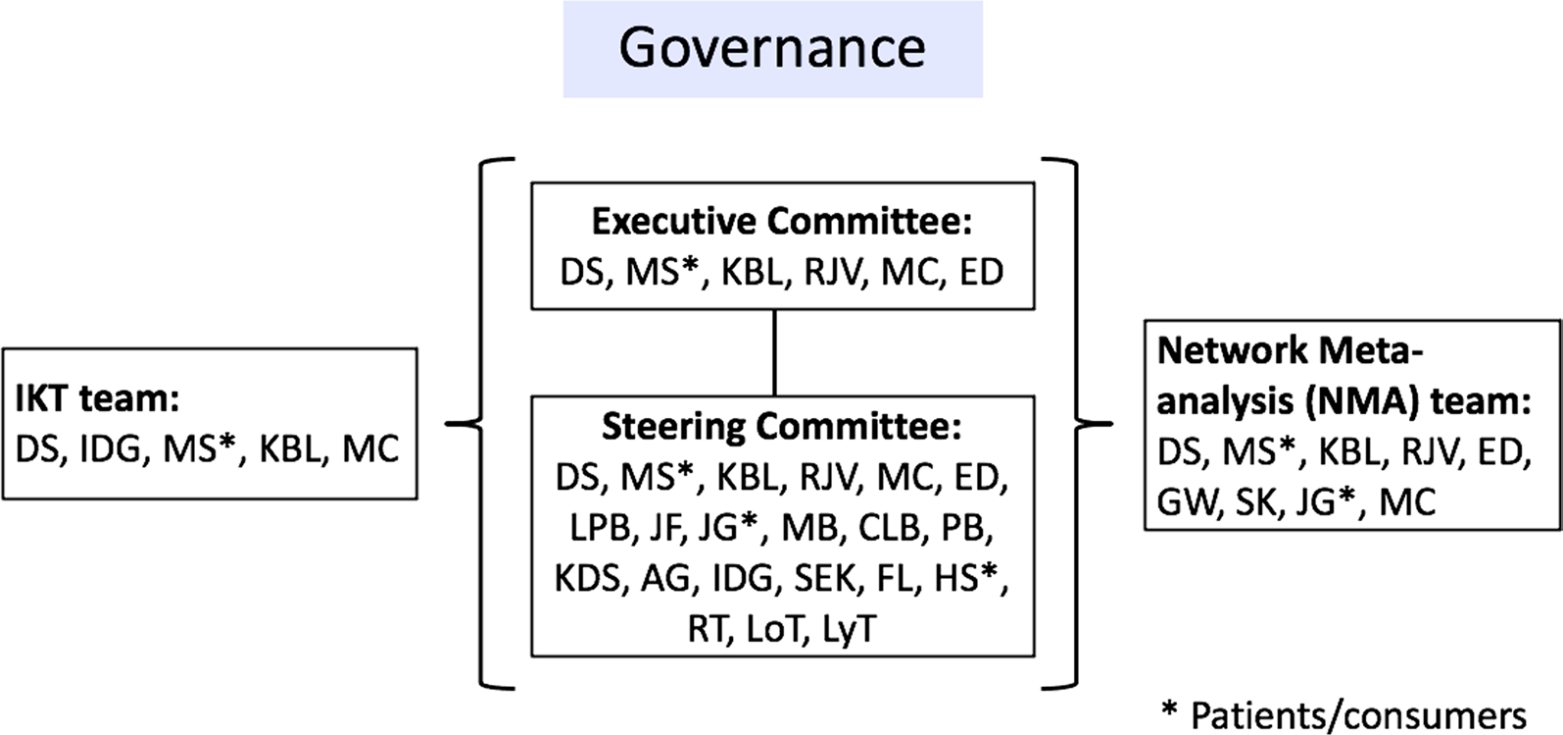
Project governance

The systematic review principal investigators (n = 4) established a project governance structure that included an executive committee, an international steering committee, the IKT team, and the network meta-analysis team (see Fig. 1). Starting in March 2022, the executive committee (DS, MS, RJV, KBL, ED), including a patient/consumer (MS) and research coordinator (MC), met every two weeks to build and maintain momentum throughout the project and ensure milestones were met. The 21-member steering committee was composed of twelve researchers, five clinician-researchers, three patients/consumers, and one graduate student. Study updates were communicated to steering committee members via monthly email updates and two virtual 1-h synchronous meetings; one prior to project launch (April 2022) and the second to share preliminary findings and discuss interpretation (February 2023). The IKT team with a patient/consumer (DS, IDG, MS, MC and KBL) was responsible for all aspects related to the evaluation of the IKT approach for the systematic review. After the review was submitted to the Cochrane Library in April 2023, the findings from this updated Cochrane Review were sent to the network meta-analysis team for further analysis (GW, SK, DS, MS, KBL, RJV, MC, ED, JG).

### Integrated knowledge translation team: positionality

Since the very first Review of Patient Decision Aids (PtDAs) published in 1999 [26], members of the research team have developed, evaluated, implemented, and used PtDAs to help people make quality health decisions. In this sixth update, our international, interdisciplinary and interprofessional team of 21 members included patients/consumers (i.e., patients, caregivers, members of the public), researchers (including post-doctoral fellows), clinician-researchers, and a graduate student. Some researchers had participated in previous updates (i.e., established relationships), while other researchers and knowledge users were joining for the first time (e.g., new relationships). Current team members represented diverse genders, career stages, knowledge user groups, spanning six countries (i.e., Australia, Canada, Chile, Denmark, United States, United Kingdom). Amongst us, research and clinical expertise included patient engagement, systematic reviews, IKT, library science, health economics, implementation science, and intersectionality. Together, we were committed to applying an IKT approach. All team members contributed to the development of the research protocol submitted to the Canadian Institutes of Health Research (CIHR) for funding. A subset of the main team, researchers (KBL, DS, IDG) and one patient partner (MS) was particularly interested in understanding and evaluating how research team members partner and engage in the systematic review research process. We summarize key components of knowledge users’ engagement in the systematic review using the ACTIVE framework: Authors and Consumers Together Impacting on eVidencE [27] (Additional file 1) and report our patient and public involvement in this study using the GRIPP2 checklist (Additional file 2) [28].

### Study participants

All team members, excluding the research coordinator, were eligible to participate in this descriptive study. The research coordinator led the recruitment, managed survey distribution, and received responses.

### Study procedures and data sources

Prior to the launch of the systematic review project, the research coordinator sent an email invitation and copy of the informed consent form to all team members. It was clearly stated that participation in the IKT evaluation was completely voluntary; agreement or refusal to participate had no impact on their status within the research team. Surveys were conducted at two time points: at baseline, before starting the systematic review, and after submitting the systematic review to the Cochrane Library. The baseline survey was administered using SurveyMonkey and took on average 9 min to complete and the follow-up survey took on average 13 min to complete. The research coordinator sent email reminders weekly for three weeks to those who had not completed the survey, according to Dillman’s schedule [29]. Additional data sources were executive and steering committee agendas and minutes and received email responses to monthly email updates which helped to provide context when interpreting the findings of the survey.

#### Baseline survey (pre): ahead of systematic review conduct

Team members who consented were invited to complete the baseline survey in April 2022 which included questions about their previous experiences with systematic reviews, PtDAs, IKT, and their demographic characteristics. Participants were asked to indicate their preferred level of involvement with each step of the systematic review process by indicating whether they wished the team to “keep me up to date on the progress (e.g., monthly emails)” or “invite me to participate in this step” (e.g., screen articles, provide feedback on the draft manuscript). The systematic review research process was listed in 12 steps: screen titles and abstracts, reconcile conflicts from title/abstract screening, screen full-text articles, reconcile conflicts from full-text screening, review interventions to verify they meet minimal definition of a PtDA, search grey literature sources, data extraction, assess risk of bias of included studies, assist with interpretation of the results of the analysis, assess Grading of Recommendations, Assessment, Development, and Evaluations (GRADE) evidence certainty ratings, discuss planning for the network meta-analysis, and provide feedback on the draft manuscript.

#### Follow-up survey (post): after submitting the systematic review for publication

**Fig. 2:**
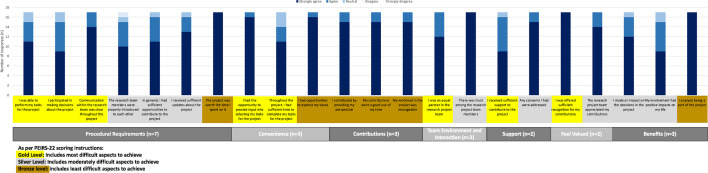
Patient engagement in research scale—PEIRS-22 (n = 17)

Once the completed systematic review was submitted to the Cochrane Library, members were invited to complete the follow-up survey in May 2023 about their degree of satisfaction with their achieved level of involvement, meaningful engagement, and experience with the IKT approach. Each survey was tailored to the participant based on their preferred level of involvement for each step provided at baseline. We used the Patient Engagement In Research Scale (PEIRS-22) to measure meaningful engagement of all team members [9]. The PEIRS-22 consists of 22 items across seven subdomains each rated on a 5-point Likert scale (5—strongly agree to 0—strongly disagree). The seven subdomains are procedural requirements, convenience, contributions, team environment and interactions, support, feel valued and benefits (see Fig. 2). The total score across 22 items is calculated for a possible range of 100 (extremely meaningful engagement) to 0 (not meaningful engagement), where higher scores indicate more meaningful engagement. Psychometric evaluation has demonstrated the PEIRS-22 had good internal consistency (ordinal alpha = 0.96), floor and ceiling effect (< 15%), structural and construct validity, and reliability (ICC_2,1_ = 0.86) [9]. Given the PEIRS-22 was focused on patient engagement on the research team and we wanted to use it to measure meaningful engagement of all team members, we changed all references to *patient partners* to *research team members* reflecting the broader composition of our team. We also revised the order of the examples in brackets for the item “I was offered sufficient recognition for my contributions (for example, payment, authorship, or gifts)” by moving authorship first to be relevant for all team members given only patient/consumer partners received an honorarium. At the end of the survey, we offered open space to allow participants to describe their experiences on the team including their perspectives on the extent to which the research process was one of co-production, and the benefits and challenges of using an IKT approach for conducting a systematic review.

### Data analysis

The research coordinator shared anonymized and aggregated data by group with the IKT team for analysis and interpretation to ensure the data could not be linked back to any participant. Quantitative data were analyzed descriptively. The PEIRS-22 data were scored in accordance with the published instructions using Microsoft Excel [30]. We reported on descriptive statistics of the total subscale scores by participant. Further, according to published instructions, we report results of each PEIRS-22 item according to the three levels of meaningful engagement in research where Gold represents aspects most difficult to achieve, Silver represents moderately difficult aspects to achieve, and Bronze represents aspects least difficult to achieve. Two team members (KBL, DS) independently performed conventional content analysis on open-text responses to the question “*To what extent is the review process truly one of co-production*” and eliciting the benefits and challenges of co-producing knowledge in this way [31]. We then met to reach consensus on the findings. Qualitative and quantitative data were integrated at the level of study design (convergent) as both were collected and analyzed simultaneously, and at the level of interpretation using a narrative approach [32].

## Results

### Participant characteristics

Twenty of twenty-one team members participated in this evaluation (95.2% response rate). Of these 20 participants, most were born in the 1950s (n = 5; 25%) and 1960s (n = 7; 35%) (Table 1). Thirteen participants (65%) were female (sex), and the same number self-identified as woman (gender). The majority reported English as their primary language (n = 15; 75%). There were 11 (55%) researchers, 3 (15%) patients/consumers, and 5 (25%) clinician-researchers. One graduate student participated, and their data were merged with those of the researchers to maintain their confidentiality. All (100%) had previous experience with PtDAs and systematic reviews. One participant reported no previous experience with IKT (Table 2). Participants were from various Countries including Australia, Canada, Chile, Denmark, the United Kingdom, and the United States of America.

**Table 1:** Participant characteristics at baseline

	n (%) n = 20 (100%)
Decade of birth	
1950’s	5 (25)
1960’s	7 (35)
1970’s	3 (15)
1980’s	4 (20)
1990’s	1 (5)
Sex	
Female	13 (65)
Male	7 (35)
Gender	
Woman	13 (65)
Man	7 (35)
Other	0 (0)
Current level of education	
Post-secondary education	1 (5)
Graduate studies	19 (95)
Primary language(s)*	
English	15 (75)
French	4 (20)
Danish	3 (15)
Spanish	1 (5)
Role that best reflects primary expertise on this research project (select one)	
Researcher	11 (55)
Patient/consumer (did not identify as researcher)	3 (15)
Clinician-researcher	5 (25)
Graduate student	1 (5)

*Participants could indicate more than one primary language

**Table 2:** Participants’ previous experiences and roles

	n (%) n = 20 (100%)	Researchers/graduate student (n = 12)	Clinician-researchers (n = 5)	Patients/consumers (n = 3)
Experience with PtDAs*				
Beginning to learn about PtDAs	2 (10)	2 (17)	0 (0)	0 (0)
Received PtDAs as an intervention in a health system	2 (10)	1 (8)	1 (20)	0 (0)
Gave PtDAs to someone making a decision	12 (60)	5 (42)	5 (100)	2 (67)
Was a participant in training to use PtDAs	7 (35)	4 (33)	1 (20)	2 (67)
Develop(ed) PtDAs	15 (75)	9 (75)	5 (100)	1 (33)
Was a participant in a research study evaluating PtDAs	8 (40)	6 (50)	2 (40)	0 (0)
Conduct(ed) research about PtDAs	16 (80)	10 (83)	5 (100)	1 (33)
Develop(ed) and/or promote(d) health policy that supports PtDAs	9 (45)	4 (33)	5 (100)	0 (0)
Other (i.e., implementation of PtDAs in clinic, participated in online training on PtDA development)	2 (10)	1 (8)	1 (20)	0 (0)
Experience with systematic reviews*				
Read or reviewed abstracts/consumer summaries of a systematic review(s)	17 (85)	12 (100)	2 (40)	3 (100)
Verified search strategies to be used in electronic databases	10 (50)	8 (67)	2 (40)	0 (0)
Screened titles and abstracts of citations	17 (85)	12 (100)	4 (80)	1 (33)
Screened full text of citations	18 (90)	12 (100)	5 (100)	1 (33)
Searched grey literature source	11 (55)	9 (75)	2 (40)	0 (0)
Extracted data into data collection forms	16 (80)	11 (92)	5 (100)	0 (0)
Assessed risk of bias of included studies	12 (60)	8 (67)	4 (80)	0 (0)
Conducted descriptive analyses of findings from eligible studies	14 (70)	10 (83)	4 (80)	0 (0)
Conducted meta-analyses	8 (40)	6 (50)	2 (40)	0 (0)
Assessed GRADE evidence ratings	9 (45)	6 (50)	3 (60)	0 (0)
Conducted network meta-analyses	3 (15)	3 (25)	0 (0)	0 (0)
Drafted a systematic review article(s)	17 (85)	11 (92)	5 (100)	1 (33)
Provided feedback on a systematic review article(s)	19 (95)	11 (92)	5 (100)	3 (100)
Co-authored a systematic review article(s)	17 (85)	11 (92)	5 (100)	1 (33)
Peer-reviewed a systematic review article(s) for a journal	16 (80)	11 (92)	4 (80)	1 (33)
Expertise with SDM and interventions to support SDM*				
Shared decision making	16 (80)	10 (83)	5 (100)	1 (33)
Patient decision aids	18 (90)	10 (83)	5 (100)	3 (100)
Decision coaching	7 (35)	5 (42)	1 (20)	1 (33)
Question prompts	6 (30)	3 (25)	2 (40)	1 (33)
Other (i.e., communication skills, decision maps, guidelines development, attended a Shared Decision-Making conference)	3 (15)	1 (8)	1 (20)	1 (33)
Experience with IKT or research co-production*				
Patients on the research team who…				
Served in a consultative or advisory capacity	16 (80)	9 (75)	4 (80)	3 (100)
Were considered equal members of the team and were involved in all or many aspects of project decision making	15 (75)	8 (67)	4 (80)	3 (100)
Served on the executive committee or steering committee	13 (65)	7 (58)	4 (80)	2 (67)
Health professionals on the research team who work clinically who…				
Served in a consultative or advisory capacity	16 (80)	10 (83)	4 (80)	2 (67)
Were considered equal members of the team and were involved in all or many aspects of project decision making	19 (95)	12 (100)	4 (80)	3 (100)
Served on the executive committee or steering committee	17 (85)	11 (92)	4 (80)	2 (67)
Health services leaders on the research team who…				
Served in a consultative or advisory capacity	10 (50)	6 (50)	2 (40)	2 (67)
Were considered equal members of the team and were involved in all or many aspects of project decision making	9 (45)	6 (50)	1 (20)	2 (67)
Served on the executive committee or steering committee	11 (55)	6 (50)	3 (60)	2 (67)

*IKT* Integrated knowledge translation, *IPDAS* International Patient Decision Aids Standards, *PtDA* Patient decision aids, *SDM* Shared decision-making

*Participants could select more than one response

### Baseline survey: preferred level of involvement in systematic review steps

Preferred level of involvement varied according to the step (Table 3). Participants’ desire to be invited to participate in specific steps ranged from (n = 20, 100%) to (n = 3, 15%), with searching the grey literature and data extraction being of least interest to all participant groups. These two steps aside, at least six researchers and two clinician-researchers wanted to be invited to participate in systematic review steps. For six of the 12 steps, no patient/consumer wanted to be invited. All participants wanted to be invited to provide feedback on the draft of the article (n = 20, 100%). Eighteen participants wanted to be invited to assist with interpretation of the results of the analysis (n = 18, 90%).

**Table 3:** Preferred highest level of involvement in systematic review steps at baseline (N = 20)

	Keep me up to date n (%)*	Invite to participate n (%)*	Invite me to participate*		
			Researchers/graduate student (n = 12)	Clinician-researchers (n = 5)	Patients/consumers (n = 3)
1. Screen titles and abstracts of citations	6 (30)	13 (65)	9 (75)	3 (60)	1 (33)
2. Reconcile conflicts from title/abstract screen	6 (30)	14 (70)	10 (83)	3 (60)	1 (33)
3. Screen full text of citations	6 (30)	13 (65)	9 (75)	4 (80)	0 (0)
4. Reconcile conflicts from full text screen	6 (30)	14 (70)	10 (83)	4 (80)	0 (0)
5. Review interventions to verify they meet minimal definition of a patient decision aid	5 (25)	12 (60)	6 (50)	4 (80)	2 (66)
6. Search grey literature sources	13 (65)	3 (15)	2 (17)	1 (20)	0 (0)
7. Extract data into data collection forms	9 (45)	7 (35)	5 (42)	2 (40)	0 (0)
8. Assess risk of bias of included studies	8 (40)	9 (45)	7 (58)	2 (40)	0 (0)
9. Assist with interpretation of the results of the analysis	1 (5)	18 (90)	11 (92)	5 (100)	2 (66)
10. Assess GRADE evidence ratings	10 (50)	8 (40)	6 (50)	2 (40)	0 (0)
11. Discuss the network meta-analysis	3 (15)	16 (80)	10 (83)	3 (60)	3 (100)
12. Provide feedback on the draft systematic review article	0 (0)	20 (100)	12 (100)	5 (100)	3 (100)

*Range of missing responses: 0–4 participants per item

### Follow-up survey: satisfaction with level of involvement and meaningful engagement

Of 20 who completed the baseline survey, 17 (85.0%) completed the follow-up survey. Overall, 16 (94.1%) participants were totally or very satisfied with the extent to which the research team engaged them in the project. One participant was satisfied (5.9%). All participants (n = 17; 100%) were satisfied with their level of involvement in assisting with interpretation of the results and the opportunity to provide feedback on the draft systematic review article (Table 4). Only researchers (range n = 1–3; 10–30%) reported dissatisfaction across six steps. Through personal communication with some team members, these responses reported disappointment with their personal level of involvement, rather than how they were engaged by the team. Three researchers were most disappointed with the network meta-analysis step, which was in the early stages when the post survey was circulated. When involved, clinician-researchers and patients were satisfied across all steps. PEIRS-22 total scores were: 13 (76.4%) participants, including all patients/consumers, reported extremely high degree of meaningfulness, two (11.8%) very high degree of meaningfulness, and two (11.8%) at a moderate level (Table 5). One to three participants reported low degrees of involvement across four subdomains: procedural requirements (n = 3; 17.6%), convenience (n = 2; 11.8%), support (n = 2; 11.8%), contributions (n = 1; 5.9%) and benefits (n = 1; 5.9%).

**Table 4:** Satisfaction with level of involvement in systematic review steps (n = 17)

Systematic review steps	Yes n (%)	No n (%)	N/A n (%)
1. Screen titles and abstracts of citations (n = 17)	14 (82)	0 (0)	3 (18)
Researchers (n = 10)	8 (80)	0 (0)	2 (20)
Clinician-researchers (n = 4)	3 (75)	0 (0)	1 (25)
Patients/consumers (n = 3)	3 (100)	0 (0)	0 (0)
2. Reconcile conflicts from title/abstract screen (n = 17)	13 (76)	1 (6)	3 (18)
Researchers (n = 10)	7 (70)	1 (10)	2 (20)
Clinician-researchers (n = 4)	3 (75)	0 (0)	1 (25)
Patients/consumers (n = 3)	3 (100)	0 (0)	0 (0)
3. Screen full text of citations (n = 17)	13 (76)	1 (6)	3 (18)
Researchers (n = 10)	7 (70)	1 (10)	2 (20)
Clinician-researchers (n = 4)	3 (75)	0 (0)	1 (25)
Patients/consumers (n = 3)	3 (100)	0 (0)	0 (0)
4. Reconcile conflicts from full text screen (n = 17)	13 (76)	0 (0)	4 (24)
Researchers (n = 10)	7 (70)	0 (0)	3 (30)
Clinician-researchers (n = 4)	3 (75)	0 (0)	1 (25)
Patients/consumers (n = 3)	3 (100)	0 (0)	0 (0)
5. Review interventions to verify they meet minimal definition of a patient decision aid (n = 17)	14 (82)	0 (0)	3 (18)
Researchers (n = 10)	8 (80)	0 (0)	2 (20)
Clinician-researchers (n = 4)	3 (75)	0 (0)	1 (25)
Patients/consumers (n = 3)	3 (100)	0 (0)	0 (0)
6. Search grey literature sources (n = 17)	11 (65)	2 (12)	4 (24)
Researchers (n = 10)	5 (50)	2 (20)	3 (30)
Clinician-researchers (n = 4)	3 (75)	0 (0)	1 (25)
Patients/consumers (n = 3)	3 (100)	0 (0)	0 (0)
7. Extract data into data collection forms (n = 17)	14 (82)	0 (0)	3 (18)
Researchers (n = 10)	9 (90)	0 (0)	1 (10)
Clinician-researchers (n = 4)	3 (75)	0 (0)	1 (25)
Patients/consumers (n = 3)	2 (67)	0 (0)	1 (33)
8. Assess risk of bias of included studies (n = 17)	13 (76)	1 (6)	3 (18)
Researchers (n = 10)	8 (80)	1 (10)	1 (10)
Clinician-researchers (n = 4)	3 (75)	0 (0)	1 (25)
Patients/consumers (n = 3)	2 (67)	0 (0)	1 (33)
9. Assist with interpretation of the results of the analysis (n = 17)	17 (100)	0 (0)	0 (0)
Researchers (n = 10)	10 (100)	0 (0)	0 (0)
Clinician-researchers (n = 4)	4 (100)	0 (0)	0 (0)
Patients/consumers (n = 3)	3 (100)	0 (0)	0 (0)
10. Assess GRADE evidence ratings (n = 17)	14 (82)	1 (6)	2 (12)
Researchers (n = 10)	8 (80)	1 (10)	1 (10)
Clinician-researchers (n = 4)	3 (75)	0 (0)	1 (25)
Patients/consumers (n = 3)	3 (100)	0 (0)	0 (0)
11. Discuss the network meta-analysis (n = 17)*	13 (76)	3 (18)	0 (0)
Researchers (n = 10)	6 (60)	3 (30)	0 (0)
Clinician-researchers (n = 4)	4 (100)	0 (0)	0 (0)
Patients/consumers (n = 3)	3 (100)	0 (0)	0 (0)
12. Provide feedback on the draft systematic review article (n = 17)	17 (100)	0 (0)	0 (0)
Researchers (n = 10)	10 (100)	0 (0)	0 (0)
Clinician-researchers (n = 4)	4 (100)	0 (0)	0 (0)
Patients/consumers (n = 3)	3 (100)	0 (0)	0 (0)

Three team members did not respond to the follow-up survey

*N/A* Not applicable selected because they did not actively participate in this step

*One participant responded “Prefer not to say”

**Table 5:**
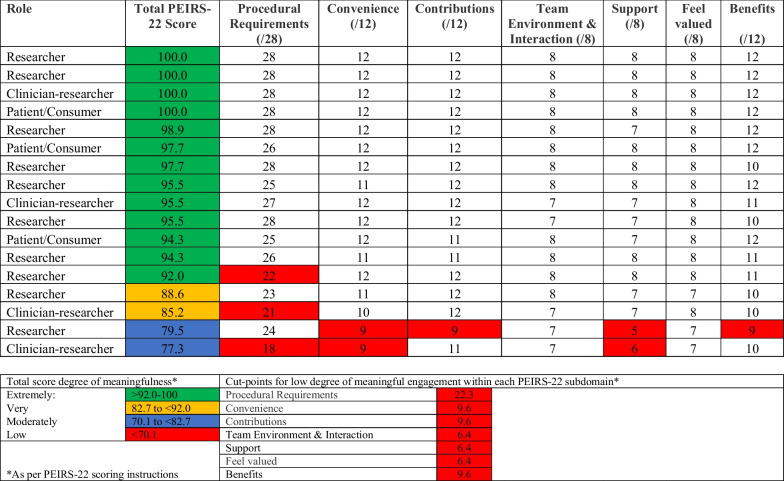
Patient engagement in research scale—PEIRS-22 (n = 17)

Most participants (range 14–17; 82–100%) strongly agreed or agreed with PIERS-22 items representing the most difficult aspects to achieve for meaningful engagement (e.g., I participated in making decisions, I had sufficient time to complete my tasks, I was an equal partner), represented by the Gold level (Fig. 2). This indicates that our team achieved an advanced level of meaningful engagement throughout this project.

### Evidence of co-production

In response to the question “To what extent is the review process truly one of co-production?” on a five-point Likert scale, all participants (100%) answered favorably, responding either to a very large extent (n = 11; 65%) or to a large extent (n = 6; 35%). One researcher summarized the process with gratitude, stating “*thank you for demonstrating to us that a co-production approach can be done and done well!”* (Researcher 1). Participants reported five main indicators that had them believe the review process was one of co-production.

#### Engagement of patient/consumer partners

Patient/consumer partners on the executive and steering committee were given the same invitations to be involved on the systematic review steps as other team members. Their involvement on these steps and participation in team meetings was reported as an indicator of co-production. One patient/consumer reported: “*I felt I was consulted as much as the team could. It was a co-production. I have been part of systematic reviews before. I felt this team truly included me*” (Patient/Consumer 1). A researcher described this by stating “*The executive team included a patient partner that attended and contributed to meetings and responded to email, plus two other patient partners on the full team”* (Researcher 2).

#### Formal invitation to participate at each step

The invitation to participate throughout the study based on team members’ desired extent and/or capacity was an indicator of co-production. This was described by a researcher who said: “*There was co-production with all team members invited to participate to the extent they wanted to participate”* (Researcher 2) and a patient partner who recognized *“the level of opportunity for involvement for everyone”* (Patient/Consumer 2). This sentiment was shared by another researcher who noted that the “o*pportunities were provided to contribute (from protocol to dissemination)*” (Researcher 3), highlighting that invitations to participate and contribute were continuous throughout the process. Two other researchers specifically highlighted that team members could participate “*based on their capacity*” (Researcher 4) and tailored “*to my schedule and interests”* (Researcher 5).

#### Feeling welcome to contribute

Participants described the team’s openness and receptivity for input from all team members. One researcher stated, “*there were lots of opportunity to provide input that was always considered*” (Researcher 6). Another researcher agreed “*the lead team was very open to opinions and to all suggestions given by the participants*” (Researcher 4).

#### Shared ownership and decision-making

Participants described a collaborative approach to decision-making. A researcher believed there was “*shared ownership of the research process and outcomes*” (Researcher 7). While another researcher said, “*the most important are that findings and decisions were discussed and agreed upon amongst the whole team*” (Researcher 8). One researcher provided examples to support this view, stating it was “*a collaborative approach to decision-making regarding, for example, primary outcomes, data interpretation*” (Researcher 7).

#### Regular and varied modes of communication

Participants recognized that the team communicated using a variety of modes which allowed them to stay updated with the project’s progress and made them feel engaged. As one researcher said, there was “*lots of communication and team meetings to discuss everything*” (Researcher 6). Similarly, a researcher remarked there was “*ongoing communication and feedback between researchers and stakeholders*” (Researcher 7). A clinician-researcher shared, “*I experienced a lot of engagement and working together in the many emails that also explained who performed which tasks*” and commented on the frequency, stating “*the number of emails along the way, which have been neither too few nor too many*” (Clinician-researcher 1).

### Benefits and challenges of using a co-production approach

Participants reported several benefits and challenges of having participated in the co-production approach. We report on the benefits first including knowledge exchange, a more authentic research process, better and more relevant outputs, managing and incorporating diverse perspectives, and personal benefit (e.g. enjoyment from being involved). Challenges included being more time and resource intensive—both to organize and to participate.

#### Knowledge exchange

There was recognition of exchanges in knowledge and expertise, and how those exchanges positively impacted the work. A researcher remarked that “*at several points patient partners had a significant effect on the way an activity was done or feedback on our work. Same with other authors*” (Researcher 2). Another researcher described how the co-production process “*allowed me to follow along with the project, even on steps where I was not actively participating. This gave me a sense of where the project was at. This approach also allowed me to contribute in the ways that were most meaningful to me*” (Researcher 5).

#### Authentic research process

Participants commented on how co-production impacted the authenticity of the research process. “*I think that using a co-production approach makes the systematic review process more authentic and ensures that the findings are accurate*” (Researcher 9). A researcher felt the process “*keeps the project patient-centered, less jargon, and more practical*” (Researcher 10). This respect for the patient perspective was echoed by a Patient/Consumer who confirmed “*I was given a chance to voice my opinions*.” (Patient/Consumer 1).

#### Better and more relevant outputs

Participants reported how a co-production process had a beneficial impact on the data interpretation: “*validates the content of this manuscript as researchers from different backgrounds and level of expertise could provide their opinion and share their experiences when analysing the data*” (Researcher 4). While another stated that “*various perspectives were captured throughout; which are important for the interpretation of the study findings”* (Researcher 1). Participants believed that a co-production approach increased the relevance and applicability of dissemination to broader audiences.

“*Increased engagement and ownership leading to increased buy-in and support for the research as well as greater uptake and implementation of the study findings—When stakeholders are involved in the research process, they can help to disseminate research findings and ensure that they reach the intended audience. This can help to maximize the impact of research and ensure that it has real-world applications and implications”* (Researcher 7).

#### Managing and incorporating diverse perspectives

Participants recognized the synergy of the varied perspectives on the team and the challenge of managing diverse views; all of which were valued. As one clinician-researcher explained, these varied perspectives offered “*many new and different angles on the content and interpretation of the study and you are not stuck in agreed frameworks and interpretations of data*” (Clinician-researcher 1). A researcher stated that the diversity of perspectives “*strengthened the research and outputs*.” One patient/consumer noted that the process allowed for “*understanding the value of the non-researcher perspectives*” (Patient/Consumer 3); while another noted that the approach “*included more diversified perspectives, and acknowledges and respects the patient perspective*” (Patient/Consumer 2).

#### Personal benefit

Participants expressed appreciation for the experience and derived personal benefit and enjoyment from being involved, as evidenced by the following comments: “*I am very satisfied with my experience on this review team*” (Patient/Consumer 1). Another Patient/Consumer shared “*I have enjoyed my experience with this team a great deal*” (Patient/Consumer 1). A researcher stated: “*This review was very interesting and the team was amazing to work with*” (Researcher 9).

When asked about challenges, participants reported the following:

#### No challenges

Three participants stated there were no challenges. A patient/consumer stated, “*for me, I don’t think there were challenges*” (Patient/Consumer 1). A researcher noted “*This was a very skilled and experienced team, so I didn't perceive significant challenges*” (Researcher 10). One researcher commended the team: “*You all made it look easy!”* (Researcher 1).

#### More time and resource intensive: to organize

Participants recognized the additional time, resources, and coordination required. Two researchers noted: “*Co-production can be a time-intensive process, requiring significant investment of time and resources to engage stakeholders and ensure their involvement throughout the research process*” (Researcher 7). While another stated “*It is extra work to ensure all people have a chance to speak and participate*” (Researcher 2). A clinician-researcher added: “*It must require far more coordination and resources to make it all play together as well as meet deadlines for multiple steps in the process.”* (Clinician-researcher 1). Although not specific to co-production*,* two researchers recognized logistical challenges of coordinating an international team given time zones, distances, and accommodating everyone’s schedules for full team meetings.

#### More time and resource intensive: to participate

Some participants reported that being part of a co-production approach may have resulted in a different type of engagement requiring more attention to each step than other team projects they have been a part of. “*From my perspective being engaged in different aspects of the project required me to stay up-to-date on what was going on. In a more traditional role, I might be more actively engaged in all phases. This ‘forces’ me to stay up to date but comes with some trade-offs (participating in all activities; larger commitment, *etc*.)”* said a researcher (Researcher 5)*.* Sometimes, competing priorities (e.g., workload) beyond the project interfered with participants engaging to a greater level. For example, “*clinicians could be busy with their clinical work, and patients may become ill and not able to participate*” (Clinician-researcher 2).

## Discussion

We aimed to evaluate research team members’ degree of meaningful engagement throughout the conduct of the Cochrane Review of PtDAs and perceptions of having been involved in an IKT approach. Most of our team members, including all patients/consumers, reported extremely meaningful engagement in the process, and they were able to describe evidence of co-production. Benefits to using the IKT approach were knowledge exchange, a more authentic research process, more relevant outputs, incorporating more diverse perspectives and personal benefit. Although some said there were no challenges, others identified IKT requiring more time and resources to organize and to participate. Our findings lead us to three points of discussion.

First, meaningful engagement of researchers and knowledge users on a diverse international systematic review team was shown to be possible. We achieved an advanced level of meaningful engagement with most team members, despite the additional time and resources required to coordinate and participate as compared to our previous experiences with updates of this review that were researcher-led. Time and resources constraints is a common challenge for co-produced research [33].

We valued any amount of time our team members could contribute at each step and acknowledged that competing professional and personal priorities influenced levels of engagement for some. Our flexible approach inviting team members to decide their preferred level of involvement in distinct systematic review steps allowed our team members to be engaged in ways they desired to be. Others have also reported such flexible approaches as a facilitator to co-produced research [34]. Of particular interest, no patient/consumer wanted to be invited to participate in six of the 12 steps and they reported no prior experience in these steps. Being aware of team members’ prior experience in specific methodological aspects of a research study may prompt leads to offer support and training as required. Our various and ongoing engagement and communication strategies throughout the life cycle of the project, including the invitation to select which steps one wanted to be involved in, may have mitigated the potential risks often incurred from power dynamics between patients/consumers and researchers [11]. Other research teams wishing to engage knowledge users should consider this approach regardless of study design to ensure knowledge users are aware of the various opportunities to be engaged in, allowing them to be engaged in the study activities that interest them most to the degree that they can and want to be.

Second, the evaluation of meaningful engagement of diverse team members in IKT research is still in the early stages. Measuring IKT or co-production is important for various reasons: to know if our engagement strategies are working as expected, to ensure we are not causing harm towards knowledge users who may not be used to being involved as partners on research teams, to learn from current processes and reflect on how we can improve next time [35]. The instrument we selected to measure meaningful engagement, the PEIRS-22, was useful in the context of a systematic review. Yet, given it was originally developed to measure meaningful engagement solely from the patient partner and family caregiver perspectives, it required minor edits to refer to all team members (e.g., patients/consumers, clinician-researchers, researchers) rather than patients alone [9, 30]. Our new question asking about their overall impression of satisfaction with their achieved level of involvement in the project provided mixed results. While most reported a high level of satisfaction, some reported they were disappointed with their personal level of participation due to competing priorities. This interpretation and response to the question did not reflect the research team’s efforts to engage them in the project. More recently, another evaluation measure has been proposed for diverse teams like ours. McLean et al. developed the RQ + 4 Co-Pro Assessment Instrument as an open access tool to evaluate co-production efforts [36]. The tool is practical in that it can be adapted on various dimensions including the (1) project that uses a co-production approach, (2) values of the team, and (3) context in which the work is being conducted.

Finally, our diverse group of researchers, clinician-researchers, patients/consumers and graduate student offered complementary expertise to successfully complete the systematic review. We valued each other’s complementary expertise through mutual trust and respect. This includes the patients/consumers’ expertise as holders of personal and experiential knowledge of facing difficult health decisions and given their prior involvement on research teams. Yet, we acknowledge that the meanings patients ascribed to concepts of expert and expertise may be more nuanced than how we considered it [37]. On numerous occasions, the patients/consumers asked questions, contributed to discussions about findings, ensured plain language in the consumer summary, keeping the collective needs of patients and caregivers at the forefront. To achieve mutual trust and respect [38], it was important for us to focus on early relationship building given we were working with some team members who were involved in previous updates and with new team members. Although the full team had not previously worked together, most knowledge users (e.g., patients/consumers, clinician-researchers) had pre-existing collaborations with one or more other team members which helped with their integration into our research team. Although there was not much latitude for team members to modify the study design and methods given this was an update of a Cochrane Collaboration review, our team worked together to co-build the research protocol, structure the grant with an IKT approach, and submitted it for funding from the CIHR. This grant proposal development process assured team members’ commitment and buy-in from the start, a facilitator to co-production identified by both Tricco et al. [15] and McLean [5].

Throughout the funded study, we had an open invitation for any steering committee member who wished to attend the bi-weekly executive committee meetings, which likely contributed to our finding that invitations to participate and contribute as an indicator of co-production were continuous throughout the process. Ultimately, we wanted our team members to have a good experience in a welcoming environment, and feel supported to be engaged to their desired level, which has been achieved by other Canadian research groups, such as the SPOR Evidence Alliance [39]. One of the main tensions we grappled with was how often to engage the full steering committee in a synchronous team meeting, with the knowledge that forums for interactions are a facilitator to co-production [15]. Planning for team meetings across time zones spanning four continents was challenging. We did so twice, prior to study launch, and again to share and interpret the findings as a team. Having limited full team meetings may explain why some participants reported that they were not properly introduced to one another. Previous research has shown that good experiences and good collaborations depend on mutual trust [6, 40]. All participants strongly agreed there was trust amongst the research team members on the PEIRS-22 item. Further, all participants reported that they enjoyed being a part of the project, and felt it was time well spent. Others specifically expressed personal benefit from being engaged with the team. This highlights that co-production can be both a rewarding and productive experience, as demonstrated by others as well [10, 38, 41].

## Strengths and limitations

Our results should be considered in view of study strengths and limitations. Strengths of the study include using the PEIRS-22 to measure meaningful engagement of all team members even though the instrument was originally developed and validated only for patient partners. Our team was diverse in age, sex and gender, geographic representation including primary language, and roles and expertise. Yet most had graduate level education with previous experience conducting systematic reviews, and patient partnered research. Hence, generalizability may be limited given this was a highly educated, experienced group. Further, given this was an update to an existing review with pre-existing collaborations for most team members. The IKT experience and challenges may be different than those encountered by teams initiating de novo reviews. There is a potential for response bias given that 20 (95%) participated in the baseline survey and 17 (85%) in the follow-up. It is possible that those with positive and/or strong views on the process may have been more likely to respond. Given this was a self-study of our team’s IKT process, responses may also be subject to social desirability bias as respondents may have provided responses to please the team. The framing of some questions may have impacted the way in which they were interpreted. For example, the research coordinator received an email indicating a participant was disappointed with their level of involvement because they had competing demands and that it was not about how the IKT approach was conducted. This led to some dissonance between how the question was worded and its interpretation. While participants were generous with their open-ended comments, a future qualitative study may provide additional, deeper insights into the IKT approach.

## Conclusion

Using an IKT approach to conduct this systematic review led to high levels of involvement by all team members including researchers, clinician-researchers, and patients/consumers. Most members indicated they were able to participate at their preferred level of involvement and were highly satisfied with the process and their level of involvement. Engaging a diverse research team composed of patients/consumers, clinician-researchers, researchers, and a graduate student in systematic review conduct aimed to ensure the relevance and potential impact of the findings. Participants indicated greater impact having been involved in an authentic research process, incorporating diverse perspectives, which they perceived resulted in better and more relevant outputs. Challenges were mostly about time, resources, and collaborating with a large group. Some wondered if there were expectations that they should be participating to a greater extent. Our findings may be helpful to other collaborative research teams with their efforts to engage patients/consumers, clinicians, and policymakers into their knowledge syntheses studies.

### Supplementary Information

**Additional file 1:** The ACTIVE framework of involvement in a systematic review.

**Additional file 2:** GRIPP2 short form.

## Data Availability

The datasets used and/or analysed during the current study are available from the corresponding author on reasonable request.

## Abbreviations

CIHR: Canadian Institutes of Health Research
GRADE: Grading of Recommendations, Assessment, Development, and Evaluations
IKT: Integrated knowledge translation
IPDAS: International Patient Decision Aids Standards
PEIRS: Patient Engagement In Research Scale
PtDA: Patient decision aids
STROBE: Strengthening the Reporting of Observational Studies in Epidemiology
