# Supplementary material for: Evaluation of an integrated knowledge translation approach used for updating the Cochrane Review of Patient Decision Aids: a pre-post mixed methods study
Source: Res Involv Engagem. 2024 Feb 9;10:21. doi: 10.1186/s40900-024-00550-w (PMC10854135; doi:10.1186/s40900-024-00550-w)
Supplement: Supplementary file 1 — Additional file 1: The ACTIVE framework of involvement in a systematic review. [file 40900_2024_550_MOESM1_ESM.docx]

**Supplemental Material 1**

**The ACTIVE framework of involvement in a systematic review**

| ***Framework Constructs*** | ***Categories*** | | | ***Key / Icon*** | |
| --- | --- | --- | --- | --- | --- |
| **Who is involved?** | Patients, carers and / or their families | | | 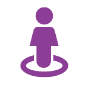 | |
|  | **🗸Patients, carers and / or their families + other stakeholders**  *Members of our team included twelve researchers, three patient/consumers, four clinician-researchers, and graduate student/post-doctoral fellows.* | | | 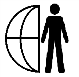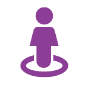 | |
|  | Other stakeholders only | | | 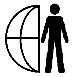 | |
| Fixed 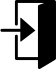 | | | | | |
| **How are people recruited?** | **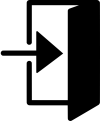**Open | | Fixed | Flexible 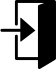 | |
|  |  |  | Flexible | 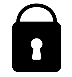 Invite | |
|  | **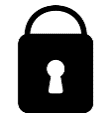**Closed | | **🗸 Invitation**  *New members were invited to diversify the group on various dimensions.* | 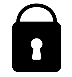 Group | |
|  |  |  | **🗸 Existing group**  *Given this is the sixth update of an established systematic review, team members from the previous updates were invited to continue their participation.* | 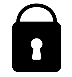 Sample | |
|  |  |  | Purposive sampling |  | |
|  | Other / Unclear | | | **?** | |
|  | | | | | |
| **What happened?**  *Approach?* | One-time | | | 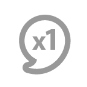 | |
|  | **🗸 Continuous**  *All team members were invited to contribute to the development of the research protocol submitted to the Canadian Institutes of Health Research for funding. Once funded*, *our project governance structure included an executive committee, an international steering committee, the IKT team, and the network meta-analysis team. The* ***executive committee*** *(DS, MS, RJV, KBL, ES) including a patient partner (MS) and research coordinator (MC) met every two weeks to discuss study progress and make decisions. The international* ***steering committee*** *was composed of the principal investigators, co-investigators, and graduate students. The same team members/knowledge users were invited to participate in all steps throughout* the entire review process. | | | 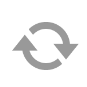 | |
|  | Combined (i.e. both one-time and continuous) | | | 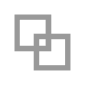 | |
|  | | | | | |
| **What happened?**  *Methods?* | **🗸 Direct interaction**  *There were two virtual meetings for international steering committee members, one prior to study launch (April 2022) and the other to share preliminary findings and discuss interpretation (February 2023). The executive committee met virtually on a biweekly basis for which there was an open invitation for any steering committee member who wished to attend. Given the ongoing pandemic at the time of the receipt of funding, we did not plan in-person meetings. Study updates were communicated to steering committee members monthly via email.* | | | **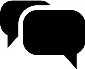** | |
|  | No direct interaction | | | **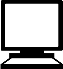** | |
|  | | | | | |
| **Stage & Level?**  **SEE BELOW FOR EXPLANATIONS** 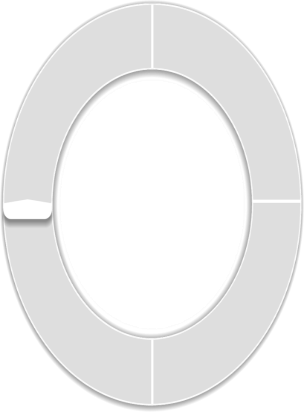 1  Develop question  2  Plan methods  3  Write & publish protocol  4  Develop search  5  Run search  6  Select studies  7  Collect data  8  Assess risk of bias  9  Analyze data  10  Interpret findings  11  Write & publish review  *12*  *Knowledge translation & impact*  **ACTIVE stages of a systematic review** |  | **🗸 Leading**  *- All team members contributed to the study protocol development and application for grant funding*  *- Shared governance structure*  *- Executive committee was composed of researchers, a patient/consumer, and research coordinator for decision-making and ensure study progress. There was an open invitation for steering committee members to attend the bi-weekly executive team meetings.*  *- Team members were invited to participate in every step of the systematic review process and provide feedback.*  *- Team members revised the manuscript for important intellectual content and granted co-authorship based on engagement throughout* | | | Lead |
|  |  | **🗸 Controlling**   - *Ahead of review conduct, all team members were invited to indicate their preferred level of involvement in every step of the review based on their interest and capacity. Some team members may have chosen to be less involved in certain steps.* | | | Control |
|  |  | Influencing | | | Influence |
|  |  | Contributing | | | Contribute |
|  |  | Receiving | | | Receive |
|  | Top & tail approach?   - No | | | 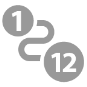 | |
