# Supplementary material for: Evaluation of an integrated knowledge translation approach used for updating the Cochrane Review of Patient Decision Aids: a pre-post mixed methods study
Source: Res Involv Engagem. 2024 Feb 9;10:21. doi: 10.1186/s40900-024-00550-w (PMC10854135; doi:10.1186/s40900-024-00550-w)
Supplement: Supplementary file 2 — Additional file 2: GRIPP2 short form. [file 40900_2024_550_MOESM2_ESM.docx]

**Supplemental Material 2**

**GRIPP2 Short Form**

| Section and topic | Item | Reported on page No |
| --- | --- | --- |
| 1: Aim | Report the aim of PPI in the study  *Together, we were committed to applying an IKT approach. A subset of the main team, researchers (KBL, DS, IDG) and one patient partner (MS) was particularly interested in understanding and evaluating how research team members, including knowledge users, partner and engage in the systematic review research process*. | Page 8 |
| 2: Methods | Provide a clear description of the methods used for PPI in the study  *The* ***executive committee*** *(DS, MS, RJV, KBL, ED) including a patient/consumer (MS) and research coordinator (MC) met every two weeks to discuss study progress and make decisions. The* ***steering committee*** *was composed of the principal investigators, co-investigators including three patients/consumers, and graduate student/post-doctoral fellow. The IKT team with a patient/consumer (DS, IDG, MS, MC and KBL) was responsible for all aspects related to the evaluation of the IKT approach for the systematic review.* | Page 9 |
| 3: Study results | Outcomes—Report the results of PPI in the study, including both positive and negative outcomes  Please refer to Table 5 and patient/consumer quotes integrated throughout the qualitative findings (p. 13-18). | Table 5  and  Pages 14-19 |
| 4: Discussion and conclusions | *Outcomes—Comment on the extent to which PPI influenced the study overall. Describe positive and negative effects*  *There are several lessons we learned that can inform our future work****.*** *Our diverse group of researchers, clinicians, patients/consumers and graduate students offered complementary expertise to successfully complete the systematic review…. we created an executive committee with a patient partner. This executive committee met bi-weekly which helped build and maintain momentum throughout the project and ensure milestones were met. Throughout the study, we had an open invitation for any steering committee member who wished to attend the bi-weekly executive committee meetings, which likely contributed to our finding that invitations to participate and contribute as an indicator of co-production were continuous throughout the process.* | Page 21-22 |
| 5: Reflections/critical perspective | Comment critically on the study, reflecting on the things that went well and those that did not, so others can learn from this experience  *There are several lessons we learned that can inform our future work****.*** Please refer to this entire discussion point for our reflections. | Page 21-22 |

PPI=patient and public involvement
